# What’s new in Emergencies, Trauma and Shock? Anesthesia, surgery and postoperative cognition

**DOI:** 10.4103/0974-2700.76816

**Published:** 2011

**Authors:** Ramesh Ramaiah

**Affiliations:** Department of Anesthesiology and Pain Medicine, University of Washington, Harborview Medical Center, Box 359724, 325 Ninth Avenue, Seattle, WA 98104-8009

Post-operative cognitive dysfunction or POCD is a post-operative memory and/or thinking impairment that has been corroborated by neuropsychological testing.[1] Postoperative cognitive dysfunction should not be confused with delirium, which is a transient and fluctuating disturbance of consciousness that tends to occur shortly after surgery,[2] whereas POCD is a more persistent problem of a change in cognitive performance. There is a general agreement that POCD is likely to be multifactorial; it remains unclear whether its occurrence is a result of surgery or anesthesia. Adverse effects of anesthesia in elder population were first described over six decades ago.[3] There is no longer any doubt that cognitive impairment occurs in some elderly patients after uncomplicated anesthesia and surgery. One of the hypotheses is that general anesthesia itself may be the cause for the development of POCD, and regional anesthesia would reduce the incidence. Several studies have investigated this issue. One of the earlier studies looking at the mental function in elderly patients following total hip replacement under either general anesthesia or regional anesthesia.[4] The authors reported that statistically significant “mental changes” (lack of orientation and amnesia for personal data) were seen in the general anesthesia group (*P*=.01) during immediate postoperative period. However, they had not done any formal neuropsychological testing on their study population. William-Russo *et al*. conducted a randomized trial to compare the effect of epidural *vs* general anesthesia on the incidence and long-term cognitive dysfunction after total knee replacement in 262 elderly patients.[5] They conclude that the type of anesthesia whether general or epidural had any effect on the magnitude or pattern of POCD. As of today, the largest study (*n*=428) conducted by Rasmussen *et al*. found no significant difference in the POCD between general anesthesia (19.7%) and regional anesthesia (12.5%) at either one week or three months following surgery.[6] When they did a *per protocol analysis* by excluding 59 patients who did not complete the study, there was a significant difference in the incidence of POCD between GA (21.2%) and EA (12.7%) after one week; however, there was no difference after three months. Although the mechanism for the development of POCD is unclear, the risk factors including increasing age, level of education, surgical duration, peri-operative infection, second operation, preoperative symptoms of mild cognitive impairment and depression etc need to be considered.[7] Development of POCD, irrespective of anesthetic technique used, indicates that the surgery may be a contributing factor. Surgical trauma and stress cause the release of neuroendocrine hormones and trigger an inflammatory response with release of cytokines that may be responsible for changes in brain function and recovery. A relationship between inflammatory mediator interleukin 6 and late functional recovery has been reported.[8] In another study, authors conclude that surgery triggers a transient neurocognitive decline in a rat model that is temporarily associated with activation of glial cells and increased proinflammatory cytokines in the hippocampus.[9] Some measures against perioperative inflammatory response may be considered as a new pathway to prevent POCD.

POCD has been subjected to extensive research both following cardiac and noncardiac surgery in the elderly population. In the published literature, very large differences can be seen in the methodology such as the test batteries utilized, the interval between sessions, the endpoints, statistical methods, and the definition of neuropsychological deficits and POCD. The study conducted in this edition on the impact of general *vs* epidural anesthesia on early post operative cognitive dysfunction following hip and knee surgery report that there may be some early post operative cognitive impairment in some subset of cognition following general anesthesia. The authors did not estimate the cognitive function at three months to see if POCD persisted beyond several months in the post operative period. Even though other studies have reported early post operative impairment of cognition in elderly population (most of them are small studies), there is no firm evidence to support general anesthesia affects learning and memory and additional research is needed to clarify the relationship between anesthesia and postoperative cognitive changes. POCD is a problem that will acquire increasing importance as the population ages, and a multidisciplinary approach involving neuroscientists, geriatricians, molecular biologists, neuropsychologists, in addition to anesthesiologists, will be needed. POCD is a multifacorial origin and it is difficult to single out the cause and additional research to investigate the methods to prevent this devastating complication is needed. At present, it is impossible to recommend an ideal anesthetic technique that will change cognitive outcome until further evidence. All available data support the concept that maintenance of adequate tissue oxygenation and hemodynamic stability, as well as a well planned anesthetic, might improve cognitive outcome in elderly patients.[10] Healthcare professionals including anesthesiologists and surgeons should inform the concerned patients and their families about the nature and risk of postoperative cognitive problems.
